# KAT5 Negatively regulates the proliferation of prostate cancer LNCaP cells via the caspase 3-dependent apoptosis pathway

**DOI:** 10.1080/19768354.2019.1644372

**Published:** 2019-08-01

**Authors:** Chul-Hong Kim, Dong Ho Lee

**Affiliations:** Department of Life Science, Chung-Ang University, Seoul, Republic of Korea

**Keywords:** KAT5, prostate cancer, LNCaP, apoptosis

## Abstract

Prostate cancer is one of the most common cancers in men over the age of sixty. Lysine acetyltransferase 5 (KAT5) is a histone acetyltransferase involved in transcriptional regulation, DNA repair, and cell signaling pathways. Previous studies have shown that KAT5 expression is reduced in the cytoplasm of the prostate cancer cell line LNCaP when exposed to androgen. Moreover, KAT5 has been reported to have a role in the molecular pathway leading to androgen-independent prostate cancer after long-term androgen deprivation therapy. Here, we showed that KAT5 expression was significantly reduced in prostate cancer tissues and cell lines by using the public databases Oncomine and Human Protein Atlas. Reduced KAT5 expression was significantly associated with high mortality in prostate cancer patients. Furthermore, KAT5 overexpression increased the level of apoptotic markers, such as cleaved-caspase 3, in LNCaP cells, thus enhancing the apoptotic death of LNCaP cells. Taken together, KAT5 induced apoptosis in prostate cancer cells via the caspase-3 pathway, indicating that KAT5 could be a gene therapy target for prostate cancer.

## Introduction

Androgen receptors (ARs) play essential roles in the development and progression of prostate cancer. In prostate cancer cells, ARs modulate the expression of proteins regulating the cell cycle, survival, and growth. AR activity appears to be closely regulated by histone acetylation in prostate cancer (He et al. 2013). Co-activators such as KAT5 (lysine acetyltransferase 5) and co-repressors such as the HDAC family bind the AR complex to facilitate or prevent the transcription of AR target genes via their respective histone acetyl transferase (HAT) or histone deacetylase (HDAC) activities (Fu et al. 2000; Korkmaz et al. 2004; Dai et al. 2007). Moreover, altered AR histone modification profiles have been reported in prostate cancer (Seligson et al. 2005).

KAT5 (lysine acetyltransferase 5) was originally identified as TIP60 (HIV-1 Tat Interactive Protein, 60 kDa) by Kamine et al. (Kamine et al. 1996). Through large-scale RNAi screening it was discovered that KAT5 was involved in an important part of the process of p53-dependent cell growth arrest (Berns et al. 2004). Recently, Gregoire et al., reported that KAT5 is a regulator of the mesenchymal-to-epithelial transition linked to the mesenchymal phenotype of MDA-MB-231 breast cancer cells (Gregoire et al. 2016). Moreover, KAT5 expression was significantly increased in clinical prostate cancer tissues and castration-resistant prostate cancer cells, with its expression level closely related to disease progression (Halkidou et al. 2003). KAT5 is an emerging candidate therapeutic target in cancer, with several inhibitors having been developed recently (Furdas et al. 2012; Farria et al. 2015); however, the functional role of KAT5 in prostate cancer has not yet been fully elucidated.

In this study, we found that KAT5 expression was significantly reduced in prostate cancer tissues and cell lines. Moreover, low levels of endogenous KAT5 in the prostate cancer cell line were closely related with the low expression of apoptosis-related proteins in the same cell line. Furthermore, ectopic KAT5 expression significantly induced the apoptotic cell death of prostate cancer cells. Here, we showed that KAT5 negatively regulates the growth of prostate cancer cells, suggesting that KAT5 could be a therapeutic candidate for prostate cancer therapy.

## Materials and methods

### Cell culture and transfection

The human prostate cancer cell line LNCaP was obtained from Korea Cell Line Bank (Seoul, Korea). LNCaP cells were grown in RPMI 1640 (Welgene, Seoul, Korea) supplemented with 10% fetal bovine serum (FBS; Welgene, Seoul, Korea), 1% penicillin–streptomycin (Welgene, Seoul, Korea) at 37°C in a humidified, 5% CO_2_ atmosphere. Cells were transfected with different plasmid DNA constructs using Lipofectamine 2000 (Invitrogen) according to the manufacturer’s instructions.

### Western blotting

The collected cells were lysed with a buffer containing 1% Triton X-100, 150 mM NaCl, 50 mM Tris-HCl (pH 7.5), 0.1% sodium dodecyl sulfate (SDS), 1% Nonidet P-40, and 1 mM PMSF by vortexing. The cell suspensions were incubated on the ice for 3 min and centrifuged at 13,000 rpm at 4°C for 10 min. The protein samples were electrophoresed on a 10% SDS-PAGE and transferred to a nitrocellulose membrane (Protran™; Whatman, Maidstone, UK). After incubation with 5% skim milk in TBST (20 mM Tris–HCl (pH 7.6), 137 mM NaCl and 0.1% Tween-20) for 1 h, the membrane was washed 3 times with TBST and incubated with antibodies against PARP-1 (1:1000, sc-56197, Santa Cruz), Caspase 3 (1:1000, #9662, Cell Signaling), Bax (1:500, #2772, Cell Signaling), Cytochrome C (1:1000, sc-13156, Santa Cruz), Bcl-w (1:500, sc-11422, Santa Cruz), Bcl-2 (1:500, sc-7382, Santa Cruz), p21 (1:1000, sc-6246, Santa Cruz), β-actin (1:1000, sc-47778, Santa Cruz), KAT5 (1:1000, sc-5725, Santa Cruz) at room temperature. Membranes were washed three times for 10 min with TBST and incubated with a 1:5000 dilution of horseradish peroxidase-conjugated anti-mouse or anti-rabbit antibodies for 1 h. Blots were washed with TBST three times and developed with the Western blotting luminol reagent (sc-2048, Santa Cruz) according to the manufacturer’s protocols.

### RNA preparation and RT-qPCR

Total RNA was isolated using TRIzol reagent (Invitrogen) according to the manufacture’s specifications. Contaminated genomic DNA was removed from 5 μg of total RNA by incubation with 10 units of RNase-free DNase I (New England Biolabs) and 2 units of RNase inhibitor (New England Biolabs) in DEPC-treated water. The reaction mixture was incubated for 1 h at 37°C and then for 10 min at 60°C. A quantitative RT–PCR (qRT-PCR) method was used to measure gene expression in cells. Oligo-dT (TAKARA, Seoul, Korea) was used as the primer in the first step of cDNA synthesis. Total RNA (1 μg) was combined with 1 μl of oligo dT, and H_2_O and then preheated at 70°C for 5 min to denature the secondary structures. The mixture was then cooled rapidly to 4°C, after which 2 μl of 10X reverse-transcriptase buffer, 10 mM DTT, and 200 units of reverse transcriptase (TAKARA, Seoul, Korea) was added to give a total volume of 20 μl. The reverse-transcriptase mix was incubated at 40°C for 1 h, after which it was stopped by heating at 94°C for 30 sec. The cDNA stock was stored at −20°C. The specificity of each of the amplified products was confirmed by melting curve analysis. For Real-Time PCR, the iQ SYBR Green PCR Supermix (Bio-Rad) and the CFX96 Real-time PCR detection system (Bio-Rad) were used to detect the Real-time quantitative PCR products of reverse-transcribed cDNA samples according to the manufacturer’s instructions.

### Cell viability assay

LNCaP cells were seeded at a density of 5 × 10^4^ cells per well in six well plates in RPMI 1640 with 1% FBS and transfected with indicated expression vectors using Lipofectamine. MTT (3-(4,5-Dimethylthiazol-2-yl)-2,5-Diphenyltetrazolium Bromide) was dissolved at 5 mg/ml in PBS solution. Two days after transfection, 400 μl of MTT solution was directly added to the medium and the cells were incubated for an additional 4 h. After removal of the medium, 4 ml of 0.04 N hydrochloric acid in isopropanol was added to each well and the optical density of the destained solution was measured on a Multiscan Go microplate reader (Waltham, MA) at a wavelength of 570 nm.

### Cell cycle analysis

LNCaP cells were trypsinized, and the resulting cell suspensions were centrifuged at 3000 rpm for 3 min. The cells were fixed for 1 h in 70% ethanol at 4°C and centrifuged at 3000 rpm for 3 min, and the pellets were washed with ice-cold PBS. Cell pellets were then resuspended in 0.1 ml of PBS containing 100 μg/ml RNase A for 1 h at 37°C. After that, 50 μg/ml of propidium iodide (Sigma-Aldrich) was added and then incubated for 15 min at RT in a dark. PI-stained cells were analyzed by BD Accuri™ C6 Plus (BD FACS, San Jose, CA). At least 10,000 cells were used for each analysis, and the results were displayed as histograms. The percentage of cell distribution in Sub-G1, G0/G1, S and G2/M phase were measured, and the results were analyzed by the BD Accuri™ C6 Plus software for cell cycle profile.

### Annexin V and propidium iodide (PI) staining for apoptosis assay

To assess the apoptosis, the cells were harvested and centrifuged at 3000 rpm for 3 min. cell proportions were detected using FITC Annexin V Apoptosis Detection Kit I (BD pharmingen, La Jolla, CA) according to the manufacturer’s instructions. The cell pellets were washed with 1X PBS and resuspended in 100 μl of Annexin V binding buffer. Cells were labeled with 5 μl of Annexin V-Alexa Fluor 488 and/or PI. After 15 min incubation in a dark, 400 μl of Annexin V binding buffer was added to wash the Annexin/PI stained cells. A minimum of 10,000 cells per sample was analyzed using BD Accuri™ C6 Plus (BD FACS, San Jose, CA).

### Cell proliferation assay

For the assay of colony forming efficiency in soft agar, transfected LNCaP cells were counted using a hemocytometer. 5 × 10^3^ cells in 1 ml growth medium containing 0.35% Noble agar (DIFCO Laboratories, Detroit, MI, USA) and G418 (800 μg/ml) were incubated in a 60 mm culture dish overlaid on 1.5 ml of 0.5% base agar medium. Cells were incubated at 37°C in a moist atmosphere of 95% air and 5% CO_2_ and 4 weeks later the number of colonies was counted after 0.005% crystal violet staining.

### Statistical analysis

Statistical analysis of variances between two different experimental groups was performed with Tukey’s *post hoc* comparison test using SPSS (Version 11.5). All experiments were repeated at least three times. The levels are considered significant at a *P* value of < 0.05 (*) very significant at a *P* value of < 0.01(**), definitely significant for at a *P* value of < 0.001(***), or not significant (n.s.).

## Results

### Low KAT5 expression in prostate cancer tissues and cell line

**Figure 1.**
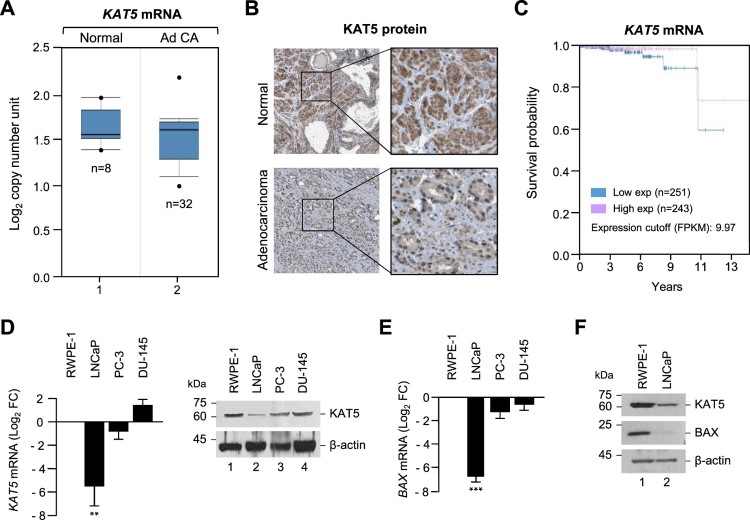
KAT5 expression was downregulated in human prostate cancer cells. (A) The gene expression data set from the Oncomine database showed that KAT5 genes were under-expressed in prostate cancer tissues (adenocarcinoma) compared with normal prostate tissues. (B) KAT5 protein expression levels in prostate cancer tissues (adenocarcinoma) and normal tissues were visualized by immunohistochemistry using anti-KAT5 antibodies. Data obtained from The Human Protein Atlas. Bar indicates 100 μm. (C) Kaplan-Meier survival curves were plotted using KAT5 gene expression levels. Data obtained from Kaplan-Meier Plotter. (D) KAT5 mRNA and protein expression were detected by RT-PCR and Western blotting in the normal cells or various cancer cells. Results represent the mean ± SEM of three biological experiments performed in triplicate. Statistical significance was determined by Student’s t-tests (***, *p* < 0.001, **, *p* < 0.01). (E) Bax mRNA expression detected by RT-PCR in the normal or various cancer cells. (F) Bax protein levels in the normal or prostate cancer cells (adenocarcinoma). KAT5 and Bax mRNA or protein levels were normalized using β-actin.

Androgen receptor (AR) co-regulators modify AR gene transactivation and prostate cell survival via acetylation, phosphorylation, ubiquitination, and sumoylation (van der Steen et al. 2013). The ubiquitously expressed acetyltransferase, KAT5, is expected to play an essential role in prostate cancer. In order to determine the possible role of KAT5 in prostate cancer, we investigated KAT5 expression levels in prostate cancer patients compared to corresponding normal individuals using the Oncomine database (normal; n = 8, prostate cancer patients; n = 32). As shown in Figure 1(A), the KAT5 expression profile was significantly lower in prostate cancer patients than in the normal group. The Human Protein Atlas database also showed that KAT5 protein expression was significantly lower in prostate cancer tissues than in normal tissues (Figure 1(B)). Moreover, the reduced KAT5 expression correlated with low survival rate in prostate cancer patients (Figure 1(C)). Next, we compared the mRNA and protein levels of KAT5 in various prostate cancer cell lines and in a normal human prostate epithelial cell line, RWPE-1. KAT5 expression was markedly lower in the human prostate cancer cell lines LNCaP and PC-3 than in the RWPE-1 cells. Interestingly, low KAT5 expression in the prostate cancer cell line (LNCaP) was closely related with low expression levels of Bax, an apoptotic marker; however, Bax protein expression levels were not affected in other prostate cell lines (PC-3 and DU-145; data not shown).

### Ectopic KAT5 expression reduces prostate cancer cell proliferation

**Figure 2.**
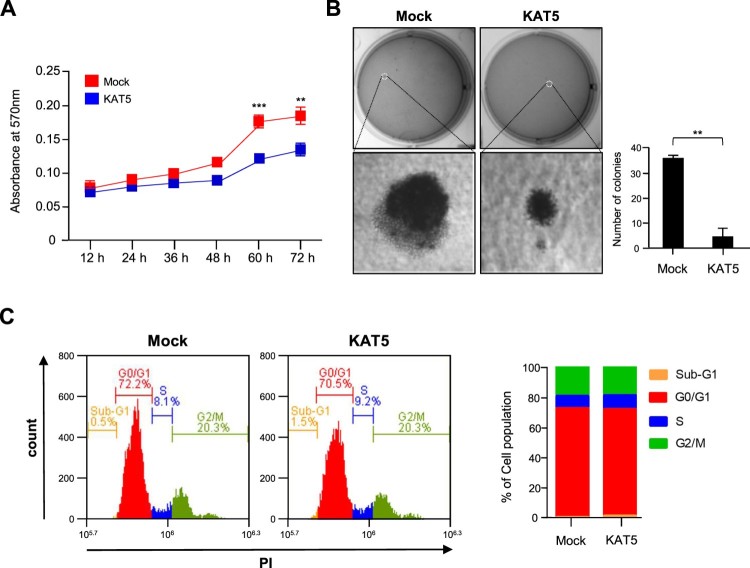
KAT5 overexpression reduced LNCaP cell proliferation. (A) Cell proliferation was examined by an MTT assay following KAT5 overexpression. (B) LNCaP cells transfected with KAT5 were plated on 0.35% agar in 6-well culture plates and the rate of colony formation was analyzed 4 weeks after incubation. Results represent the mean ± SEM of three biological experiments performed in triplicate. Statistical significance was determined by Student’s t-tests (***, *p* < 0.001, **, *p* < 0.01). (C) Flow cytometry using propidium iodide (PI) staining was performed to analyze the cell cycle in KAT5-transfected cells. The red arrow indicates the cell population (M5) with a higher chromatin content than normal transfected cells was performed. The red arrow indicates cell population (M5) with higher contents of chromatin than normal cells.

To investigate the effect of KAT5 expression on prostate cancer cell proliferation, plasmids containing the full length KAT5 gene were transfected into the human prostate cancer cell line, LNCaP. KAT5 overexpression reduced the cellular proliferation rate compared to the vehicle-transfected control group. Colony forming ability was severely reduced in the KAT5-overexpressing cells (Figure 2(A and B)), with the number of colonies significantly reduced by KAT5 overexpression in LNCaP cells. However, the cell cycle was not affected by KAT5 overexpression (Figure 2(C)).

### KAT5 induces the apoptotic death of prostate cancer cells

**Figure 3.**
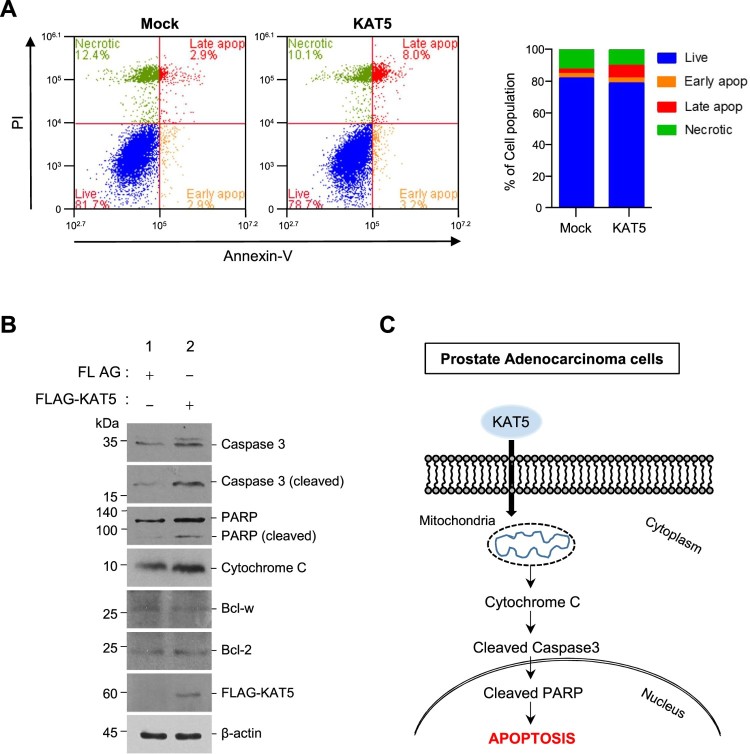
KAT5 increased apoptotic LNCaP cell death. (A) Forty-eight hours after transfection with the indicated plasmid, cells were subjected to propidium iodide (PI) or PI/Annexin-V double staining and FACS analysis was performed. (B) After cells were transfected with plasmids encoding FLAG or FALG-tagged KAT5, the expression of apoptosis-related proteins (cytochrome C, caspase 3, PARP, Bcl-2, Bcl-w) was confirmed by western blotting with β-actin as a loading control. (C) Schematic diagram of the molecular mechanism of the KAT5-induced apoptosis pathway in LNCaP prostate adenocarcinoma cells. KAT5 expression increases cytochrome C release and the activation of caspase 3 and cleavage poly-ADP-ribose polymerase (PARP), leading to apoptosis.

Since the reduced growth of prostate cancer cells was not due to changes in the cell cycle, we examined apoptotic cell death in LNCaP cells following KAT5 overexpression. Staining LNCaP cells with PI and Annexin-V revealed that both early and late apoptotic populations were significantly enhanced by KAT5 overexpression (Figure 3(A)). Moreover, the levels of apoptotic proteins such as cleaved caspase-3, cleaved PARP, and cytosolic cytochrome C increased by approximately 4.3-, 2.7-, and 1.6-fold following ectopic KAT5 expression in LNCaP cells. However, the levels of pro-survival family members (Bcl2, Bcl-w) was not affected by KAT5 overexpression (Figure 3(B)).

## Discussion

The lysine acetyltransferase KAT5 is a member of a large superfamily of epigenetic regulatory proteins that play a number of important cellular roles and are emerging as candidate targets for cancer therapy (Farria et al. 2015). Therefore, we examined the function of KAT5 in prostate cancer cells. Ectopic KAT5 expression enhanced the expression of proapoptotic proteins including Bax, cleaved caspase-3, cytosolic cytochrome c, and PARP, thus increasing the early and late apoptotic populations of prostate cancer cells (Figure 3). Our results clearly demonstrated that endogenous KAT5 levels are reduced in aggressive prostate cancer tissue (Human Protein Atlas data and Oncomine database; Figure 1).

Although KAT5 overexpression in LNCaP cells reduced cellular proliferation and colony formation, KAT5 did not slow the cell cycle (Figure 2). Under genotoxic stress conditions, KAT5 can regulate the cell cycle at the intra-S-phase checkpoint alongside ATM kinase (Ortega-Atienza et al. 2015); however, ectopic KAT5 expression did not affect cell cycle regulation in prostate cancer cells. Instead, KAT5 expression significantly increased Bax, cleaved caspase-3, cleaved PARP, and cytosolic cytochrome C levels via an intrinsic apoptosis pathway (Figure 3(C)). In particular, KAT5 plays a crucial role in p53-dependent apoptosis by specifically acetylating lysine 120 (K120) of the p53 DNA-binding domain; however, p53 regulates both cell-cycle arrest and apoptosis depending on the cellular context (Tang et al. 2006). The corresponding acetylation-defective tumor mutant (K120R) abrogates p53-mediated apoptosis, but not growth arrest in cancer cells (Tang et al. 2006), suggesting that KAT5 induces the death of prostate cancer cells via the p53-mediated intrinsic apoptosis pathway. Since p53 levels were not evaluated in this study, future investigations should elucidate whether p53 is involved in KAT5-induced prostate cancer cell death.

Histone deacetylases (HDACs), such as HDAC1, 2, 3 and 4, are highly expressed in prostate cancer tissues and their functions are over-activated. In patients with prostate cancer, high HDAC expression and hyperactivation were correlated with poor prognosis. HDAC overexpression results in accelerated proliferation, delayed differentiation, and aggressive phenotype in prostate cancer cells (Halkidou et al. 2004; Weichert et al. 2008). Consequently, HDAC function has been considered a potential therapeutic target for prostate cancer treatment. Clinical studies with HDAC inhibitors have shown significantly decreased levels of prostate-specific antigens (PSAs) in some patients (Munster et al. 2009).

Here, we found that KAT5 proteins were barely detectable in the prostate cancer cell line and prostate cancer tissues, similar to the pattern observed in a breast cancer cohort study (McGuire et al. 2019). In contrast, KAT5 is significantly overexpressed in malignant pleural mesothelioma (MPM) (Cregan et al. 2016). Although, much remains to be elucidated regarding the molecular mechanisms of KAT5 in prostate cancer initiation and progression, the correlation of low KAT5 expression with a higher chance of tumor aggressiveness suggest that KAT5 is an attractive biomarker of prostate cancer. Thus, our results may provide a useful drug target for the treatment of patients with prostate cancer.

## References

[CIT0001] Berns K, Hijmans EM, Mullenders J, Brummelkamp TR, Velds A, Heimerikx M, Kerkhoven RM, Madiredjo M, Nijkamp W, Weigelt B, et al. 2004 A large-scale RNAi screen in human cells identifies new components of the p53 pathway. Nature. 428:431–437. doi: 10.1038/nature02371 15042092

[CIT0002] Cregan S, McDonagh L, Gao Y, Barr MP, O'Byrne KJ, Finn SP, Cuffe S, Gray SG. 2016 KAT5 (Tip60) is a potential therapeutic target in malignant pleural mesothelioma. Int J Oncol. 48:1290–1296. doi: 10.3892/ijo.2016.3335 26780987

[CIT0003] Dai Y, Ngo D, Forman LW, Qin DC, Jacob J, Faller DV. 2007 Sirtuin 1 is required for antagonist-induced transcriptional repression of androgen-responsive genes by the androgen receptor. Mol Endocrinol. 21:1807–1821. doi: 10.1210/me.2006-0467 17505061 PMC3839341

[CIT0004] Farria A, Li W, Dent SYR. 2015 KATs in cancer: functions and therapies. Oncogene. 34:4901–4913. doi: 10.1038/onc.2014.453 25659580 PMC4530097

[CIT0005] Fu MF, Wang CG, Reutens AT, Wang J, Angeletti RH, Siconolfi-Baez L, Ogryzko V, Avantaggiati ML, Pestell RG. 2000 P300 and p300/cAMP-response element-binding protein-associated factor acetylate the androgen receptor at sites governing hormone-dependent transactivation. J Biol Chem. 275:20853–20860. doi: 10.1074/jbc.M000660200 10779504

[CIT0006] Furdas SD, Kannan S, Sippl W, Jung M. 2012 Small molecule inhibitors of histone acetyltransferases as epigenetic tools and drug candidates. Arch Pharm (Weinheim). 345:7–21. doi: 10.1002/ardp.201100209 22234972

[CIT0007] Gregoire JM, Fleury L, Salazar-Cardozo C, Alby F, Masson V, Arimondo PB, Ausseil F. 2016 Identification of epigenetic factors regulating the mesenchyme to epithelium transition by RNA interference screening in breast cancer cells. BMC Cancer. 16:700. doi: 10.1186/s12885-016-2683-5 27581651 PMC5006536

[CIT0008] Halkidou K, Gaughan L, Cook S, Leung HY, Neal DE, Robson CN. 2004 Upregulation and nuclear recruitment of HDAC1 in hormone refractory prostate cancer. Prostate. 59:177–189. doi: 10.1002/pros.20022 15042618

[CIT0009] Halkidou K, Gnanapragasam VJ, Mehta PB, Logan IR, Brady ME, Cook S, Leung HY, Neal DE, Robson CN. 2003 Expression of Tip60, an androgen receptor coactivator, and its role in prostate cancer development. Oncogene. 22:2466–2477. doi: 10.1038/sj.onc.1206342 12717424

[CIT0010] He W, Zhang MG, Wang XJ, Zhong S, Shao Y, Zhu Y, Shen ZJ. 2013 KAT5 and KAT6B are in positive regulation on cell proliferation of prostate cancer through PI3K-AKT signaling. Int J Clin Exp Patho. 6:2864–2871. PMC3843266 24294372

[CIT0011] Kamine J, Elangovan B, Subramanian T, Coleman D, Chinnadurai G. 1996 Identification of a cellular protein that specifically interacts with the essential cysteine region of the HIV-1 tat transactivator. Virology. 216:357–366. doi: 10.1006/viro.1996.0071 8607265

[CIT0012] Korkmaz CG, Fronsdal K, Zhang Y, Lorenzo PI, Saatcioglu F. 2004 Potentiation of androgen receptor transcriptional activity by inhibition of histone deacetylation - rescue of transcriptionally compromised mutants. J Endocrinol. 182:377–389. doi: 10.1677/joe.0.1820377 15350180

[CIT0013] McGuire A, Casey MC, Shalaby A, Kalinina O, Curran C, Webber M, Callagy G, Holian E, Bourke E, Kerin MJ, et al. 2019 Quantifying Tip60 (Kat5) stratifies breast cancer. Sci Rep-Uk. 9. 10.1038/s41598-019-40221-5 PMC6405843 30846725

[CIT0014] Munster PN, Marchion D, Thomas S, Egorin M, Minton S, Springett G, Lee JH, Simon G, Chiappori A, Sullivan D, et al. 2009 Phase I trial of vorinostat and doxorubicin in solid tumours: histone deacetylase 2 expression as a predictive marker. Br J Cancer. 101:1044–1050. doi: 10.1038/sj.bjc.6605293 19738609 PMC2768109

[CIT0015] Ortega-Atienza S, Green SE, Zhitkovich A. 2015 Proteasome activity is important for replication recovery, CHK1 phosphorylation and prevention of G2 arrest after low-dose formaldehyde. Toxicol Appl Pharmacol. 286:135–141. doi: 10.1016/j.taap.2015.03.018 25817892 PMC4458209

[CIT0016] Seligson DB, Horvath S, Shi T, Yu H, Tze S, Grunstein M, Kurdistani SK. 2005 Global histone modification patterns predict risk of prostate cancer recurrence. Nature. 435:1262–1266. doi: 10.1038/nature03672 15988529

[CIT0017] Tang Y, Luo JY, Zhang WZ, Gu W. 2006 Tip60-dependent acetylation of p53 modulates the decision between cell-cycle arrest and apoptosis. Mol Cell. 24:827–839. doi: 10.1016/j.molcel.2006.11.021 17189186

[CIT0018] van der Steen T, Tindall DJ, Huang HJ. 2013 Posttranslational Modification of the Androgen Receptor in Prostate Cancer. Int J Mol Sci. 14:14833–14859. doi: 10.3390/ijms140714833 23863692 PMC3742275

[CIT0019] Weichert W, Roske A, Gekeler V, Beckers T, Stephan C, Jung K, Fritzsche FR, Niesporek S, Denkert C, Dietel M, et al. 2008 Histone deacetylases 1, 2 and 3 are highly expressed in prostate cancer and HDAC2 expression is associated with shorter PSA relapse time after radical prostatectomy. Br J Cancer. 98:604–610. doi: 10.1038/sj.bjc.6604199 18212746 PMC2243142

